# Evaluating novel methods of enhancing the impact of financial incentives on household nutrition in developing nations – authors’ reply

**DOI:** 10.1016/j.lansea.2024.100399

**Published:** 2024-04-04

**Authors:** Poppy A.C. Mallinson, Judith Lieber, Sanjay Kinra, Arindam Debbarma, Helen L. Walls, Santhi Bhogadi, Srivalli Addanki, Richa Pande, Anura V. Kurpad, Nanda K. Kannuri, Shilpa Aggarwal, Bharati Kulkarni, Eric A. Finkelstein, Sarang Deo

**Affiliations:** a London School of Hygiene & Tropical Medicine, London, WC1E 7HT, UK; b Indian School of Business, Hyderabad, 500111, India; c University of Minnesota, Minneapolis, MN, 55455, USA; d Indian Institute of Public Health, Hyderabad, 500033, India; e National Institute of Nutrition, Indian Council of Medical Research, Hyderabad 50007, India; f St John's Medical College, Bengaluru, 560034, India; g Duke-National University of Singapore Medical School, 169857, Singapore

In our study^1^ published in *The Lancet Regional Health — Southeast Asia*, we used a cluster randomised trial design to evaluate the effectiveness of a financial incentive intervention for increasing purchase of fruits and vegetables in a rural context in southern India. In this Correspondence, we reply to the concerns raised by Krishnan and colleagues.^2^ While our methodology has numerous advantages, we concur with Krishnan and colleagues^2^ that its primary aim is not to understand ‘how’ an intervention works or ‘which factors’ influence its effectiveness. To address these aims, we conducted a process evaluation in parallel which will be published shortly.

Krishnan and colleagues^2^ raise an interesting point about the potential role of different household decision-making models in the success of the intervention. Our in-depth interviews with people implementing and receiving the subsidies confirm that the recipients were mostly women (with men “very rarely” partaking). This is likely because the subsidies were provided at the market during shopping hours, and food shopping is primary done by women in this setting (as indicated in our formative research).^3^ We agree that it is relevant to consider whether the intervention might act differently in settings where men also partake in household food purchasing. We urge other researchers and programme developers to consider such contextual factors when planning interventions for their own settings. We hope that our process evaluation results will help to elucidate aspects of this process.

The narrow geographic scope of our study (one district in Telangana State) is another limitation, which we have acknowledged in the manuscript. Whilst the study district, Ranga Reddy, is urbanised in part, the study villages are far from the city (30–65 km away) and define themselves as rural, with over one third of adults working in agriculture. In this respect they lie within the rural continuum of India and represent many communities in the region (albeit not the extremely rural or remote settings). We agree with Krishnan and colleagues^2^ that our intervention would perform differently in settings without any fruit and vegetable retailers, and hope our work inspires the development of interventions for more diverse geographies.

Finally, Krishnan and colleagues^2^ commented on the lack of objective health outcome measures in our study, especially for children. Our study focussed on demonstrating feasibility of financial incentives and their effects on total household ‘purchase’ of fruit and vegetables, as this is a necessary first step for increased consumption and improved health outcomes. We hope this will help make the case for larger, longer-term studies examining nutritional and health impacts. Importantly, such designs could include assessment of intrahousehold distribution of health benefits. Our process evaluation will also provide some formative evidence on these issues.

## Contributors

PACM, SD, JL and SK contributed to ideation of the manuscript. PACM wrote the first draft. All authors provided comments on the draft and approved the final manuscript.

## Declaration of interests

This research was funded by the Drivers of Food Choice (DFC) Competitive Grants Program, which is funded by the UK Government's Department for International Development and the Bill & Melinda Gates Foundation, and managed by the University of South Carolina, Arnold School of Public Health, USA; however, the views expressed do not necessarily reflect the UK Government's official policies. SK receives research funding from Medical Research Council UK, University of Chiang Mai Thailand, and Ministry of AYUSH Gov of India. PM receives research funding from Medical Research Council UK and Ministry of AYUSH Gov of India. JL receives research funding from Medical Research Council, UK. HW receives research funding from Public Health England, the Health Services Research Initiative, and Helen Keller International, and has received honoraria from University of the Western Cape and ANH (Agriculture, Nutrition, & Health) Academy. All authors declare no conflicts of interest.
